# Towards a neurobiological understanding of pain in chronic pancreatitis: mechanisms and implications for treatment

**DOI:** 10.1097/PR9.0000000000000625

**Published:** 2017-10-25

**Authors:** Søren S. Olesen, Theresa Krauss, Ihsan Ekin Demir, Oliver H. Wilder-Smith, Güralp O. Ceyhan, Pankaj J. Pasricha, Asbjørn M. Drewes

**Affiliations:** a Department of Gastroenterology and Hepatology, Centre for Pancreatic Diseases & Mech-Sense, Aalborg University Hospital, Aalborg, Denmark; b Clinical Institute, Aalborg University, Aalborg, Denmark; c Department of Surgery, Klinikum rechts der Isar, Technische Universität München, Munich, Germany; d Department of Anaesthesiology, Pain and Palliative Medicine, Radboud University Nijmegen Medical Centre, Nijmegen, the Netherlands; e Department of Medicine, Johns Hopkins School of Medicine, Baltimore, MD, USA

**Keywords:** Chronic pancreatitis, Pain, Mechanisms, Treatment

## Abstract

**Introduction:**

Chronic pancreatitis (CP) is a disease characterized by inflammation of the pancreas resulting in replacement of the normal functioning parenchyma by fibrotic connective tissue. This process leads to progressively impairment of exocrine and endocrine function and many patients develop a chronic pain syndrome.

**Objectives:**

We aimed to characterize the neurobiological signature of pain associated with CP and to discuss its implications for treatment strategies.

**Methods:**

Relevant basic and clinical articles were selected for review following an extensive search of the literature.

**Results:**

Pathophysiological changes in the peripheral (pancreatic gland) and central nervous system characterize the pain syndrome associated with CP; involved mechanisms can be broken down to 3 main branches: (1) peripheral sensitization, (2) pancreatic neuropathy, and (3) neuroplastic changes in the central pain pathways. Disease flares (recurrent pancreatitis) may accelerate the pathophysiological process and further sensitize the pain system, which ultimately results in an autonomous and self-perpetuating pain state that may become independent of the peripheral nociceptive drive. These findings share many similarities with those observed in neuropathic pain disorders and have important implications for treatment; adjuvant analgesics are effective in a subset of patients, and neuromodulation and neuropsychological interventions may prove useful in the future.

**Conclusion:**

Chronic pancreatitis is associated with abnormal processing of pain at the peripheral and central level of the pain system. This neurobiological understanding of pain has important clinical implications for treatment and prevention of pain chronification.

## Abstract 2

We summarize the evidence for a neurobiological understanding of pain in patients with chronic pancreatitis and discuss its potential impact on prevention and treatment.

## 1. Introduction

The prevalence of chronic pancreatitis (CP) varies between studies. It has recently been estimated to be 120 to 140/100.000 and seems to be increasing but with some regional differences.^51^ It is also likely that many patients with, eg, abdominal pain, diarrhea, and malnutrition—without diagnostic classification—in reality suffer from CP. Even though excess alcohol intake is still a major risk factor for CP, recent data suggest that only about half of the patients have alcoholic pathogenesis.^87^ However, patients with alcoholic etiology often have more complications, are more frequently hospitalized, and therefore often stigmatize the patient group as a whole.^86^ The disease is characterized by inflammation of the pancreas that results in replacement of the parenchyma by fibrotic connective tissue. This leads to progressive exocrine and endocrine pancreatic insufficiency and a variety of local and systemic complications, where pain is the most important.^54^ Being present in up to 90% of the patients,^63^ CP pain significantly increases the morbidity and reduces the life expectancy of affected patients,^87^ and recent research has shown that pain is the most important factor to explain the reduced life quality and increased health resource utilization associated with the disease.^61,62^ Clinically, the early stage of CP is typically dominated by pain attacks associated with recurrent episodes of pancreatitis and local or systemic complications, whereas in the advanced stage, pain is typically more constant.^54^ Previously, it was believed that pain decreased over the time course of the disease,^1^ but more recent studies have demonstrated that this is not the case in most patients.^48^ In general, pain treatment is difficult and often neglected even in specialist centers, and this is partly explained by a lack of understanding of the pain pathogenesis. It has been the common belief that increased pressure in the pancreatic tissue and/or ductal system could explain the pain in most patients,^26^ but newer studies have not shown a relation between the microstructural or macrostructural findings (as characterized by different imaging modalities) and the pain characteristics.^31,79^ An alternative explanation research for more than a decade has indicated that the pain in many cases has a neuropathic component, with evidence of peripheral neural sensitization and nerve destruction following inflammation and fibrosis.^17,24^ Hence, experimental and human studies have provided evidence for pancreatic neuropathy and neuroplasticity at both the peripheral (pancreatic gland) and the central level of the sensory system, which to a high degree resemble that seen in neuropathic pain disorders. Along this line, pregabalin, a drug that has shown its effectiveness for neuropathic pain, was shown to be effective in patients with painful CP in a randomized placebo-controlled trial.^55^ However, pain due to, eg, complications of the disease and adverse effects to treatment is also frequent and must not be overlooked as an additional source of pain.^64^ Although the disease is still difficult to treat, the improved understanding and recent mechanistic orientated research has led to useful guidelines on how to approach treatment of CP pain in the clinical settings with the major aim to improve the patient's suffering and quality of life. In the present review, we provide an overview of the neurobiological understanding of pain in CP and highlight its implications for treatment.

## 2. Mechanisms of pancreatic neuropathy

Pancreatic neuropathy is still a mystery in current visceral pain research; pinpointing the “weak spot” for efficient intervention poses quite a challenge for the treating physicians and researchers, as pancreatic neuropathy is not limited to the organ itself. Pain originating from the pancreas initiates a chain reaction of neuronal alterations, working all the way up from peripheral to spinal and supraspinal levels, eventually culminating in cerebral reorganization. There are various ways to adequately classify pancreatic neuropathy, depending on location of emergence and quality of pain. As different organ systems are involved, one can distinguish between “peripheral” (pancreatic) and “central” (spinal and supraspinal) processes. Also, the origin of pain can be traced back to a direct “nociceptive” stimulation of nerve endings or a “neuropathic” impairment of nerves during the evolution of CP. Transitions are, however, fluid and pancreatic neuropathy involves all components in most patients. For simplicity, breaking down pathological processes leave us with 3 main “branches” of pancreatic neuropathy^14^:

(1) Peripheral nociception and sensitization.
(2) Peripheral pancreatic neuropathy.
(3) Central neuroplasticity.

### 2.1. Peripheral nociception and sensitization: the root of all evil?

**Figure 1.**
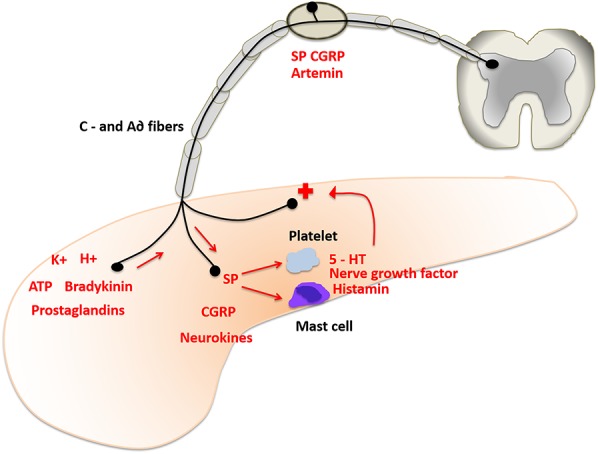
During inflammation of the pancreas, neurons respond to chemical agents, such as H+, K+, bradykinin, ATP, prostaglandins, and other inflammatory molecules, that are released following cellular damage. Substance P (SP), calcitonin gene–related peptide (CGRP), and neurokinins are transported antegrade to activate mast cells and platelets. These release serotonin (5-HT), nerve growth factor, and histamine, which again activate the sensory afferents.

Although the individual perception of pain is a product of cerebral processing,^47^ peripheral nociceptors are the origin of a frequency-encoded warning system that alerts the body to possibly hazardous stimuli.^25^ Under physiological circumstances, pain is conducted by unmyelinated C-fibers and thinly myelinated A∂ fibers, whose receptors are usually silent and unresponsive in the absence of adequate stimulation.^67^ In the course of disease, however, afferent neuron terminals respond to chemical agents such as H+, K+, bradykinin, ATP, inflammatory molecules, and trypsin that are released following cellular damage^24^ (Fig. 1). Named mediators not only spark the emergence of action potentials in local nociceptive receptors but also influence their level of activity by inducing a cascade-like liberation of other pain-promoting factors ultimately resulting in sensitization of the pancreatic sensory neurons—a process known as *peripheral sensitization*.

An examination of peripheral sensitization in CP begins with the question of whether there is evidence of increased pancreatic nociceptor excitability. In what is now one of the most validated models of CP (induced by intraductal application of trinitrobenzene sulfonic acid), Xu et al.^84^ first demonstrated that pancreas-specific nociceptors (labeled by retrograde tracers injected into the pancreas) show multiple electrophysiological changes indicating a profound increase in spontaneous activity and excitability. To further examine the molecular basis of these changes, the investigators examined voltage-gated K+ (Kv) channels that play a fundamental role in dampening neuronal excitability. What they found was that a particular Kv current, called A-type (IA), is markedly reduced in pancreatic nociceptors in animals with CP, indicating at least one mechanism responsible for the increased excitability. At the central end of the primary afferent neuron, the electrical signal is “handed off” to second-order spinal neurons via glutamate under ordinary conditions. With increasing severity of the noxious signaling, several peptides including substance P, calcitonin gene–related peptide (CGRP), and brain-derived neurotrophic factor are also involved and have been shown to be upregulated in CP models.^41,81^ An increase in spontaneous firing from dorsal root ganglia would be expected to lead to increased expression and release of these peptides, accounting in part for the increased afferent drive to central structures. In accordance with this, blockade of substance P, CGRP, and brain-derived neurotrophic factor by intrathecal antagonists can attenuate pain behavior in rats with CP.^41,52^

The effects of an increase in baseline excitability of nociceptors in CP is amplified by sensitization of “trigger” currents—small currents evoked by changes in the environment—which if sufficiently strong can depolarize the membrane to the threshold required to evoke full-blown action potentials. Thus, not only is the threshold decreased but also the currents that push the neuronal membrane to threshold are enhanced.^83^ The key molecules triggering fluctuations in membrane potentials belong to the transient receptor potential (TRP) family of ion channels. The TRP subunits that are especially enriched in nociceptive neurons, such as TRP vanilloid 1 (TRPV1), TRPV4, and TRP Ankyrin 1 (TRPA1), have shown to be involved in pancreatic inflammation, peripheral sensitization, and pain.^10^

**Figure 2.**
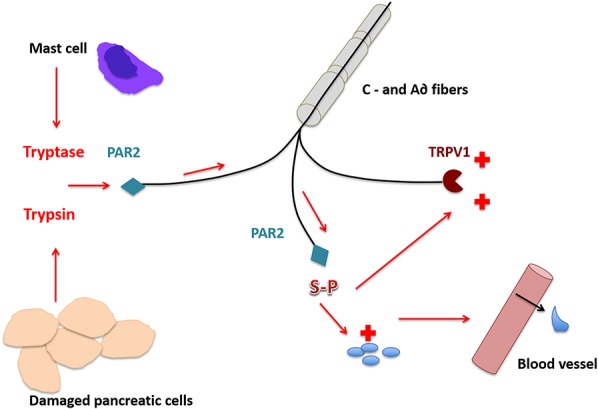
During pancreatic flares, trypsin may activate proteinase activated receptor 2 (PAR-2) on nociceptors and sensitize the response of transient receptor potential vanilloid (TRPV1) active fibres. These produce substance P (SP), the key molecule in “neurogenic inflammation” that is antegrade transported to the tissue and activates blood cells and vessels.

Probably, the most important channel responsible for trigger currents is the vanilloid receptor, TRPV1, which responds to increases in temperature and acid concentrations, amongst other factors. The role of TRPV1 in the pathogenesis of pain in CP was conclusively demonstrated in the TNBS model, in which a 4-fold increase in evoked TRPV1 currents was seen along with significant upregulation of protein and mRNA TRPV1 expression in pancreatic nociceptors. Further, systemic administration of the TRPV1 antagonist SB-366791 markedly reduced pain behavior in rats with CP (but not in control animals, suggesting a specific role in the sensitized but not the healthy state).^85^ The TRPV1 molecule may also be theoretically involved in increased pain sensation in patients who experience pancreatitis “flares,” perhaps via a mechanism involving trypsin that may be released during such episodes (Fig. 2). Trypsin is a serine protease that not only cleaves proteins but can also activate specific receptors such as proteinase activated receptor 2 (PAR-2) on nociceptors. When injected into the pancreas directly in rats, it induces a behavioral pain response that can be inhibited by desensitization of the PAR-2 receptor, suggesting an effect of pain independent of its other capabilities to injure pancreatic tissue.^72^ Activation of PAR-2 enhances capsaicin-evoked release of the pro-nociceptive neurotransmitter, CGRP, and spinal nociception, suggesting that trypsin is capable of sensitizing TRP1 via this receptor.^40^

TRPV4, another subunit of the TRP family of ion channels, responds to changes in osmotic pressure and increased temperature. Although TRPV4 has long been thought to play a subordinate role compared to TRPV1, recent studies suggest that sensitization of TRPV4 by proinflammatory mediators causes hyperalgesia to mechanical stimuli.^36^ The intraductal application of TRPV4 agonists in mice has led to increased Fos expression in spinal neurons, reflecting an increased activity of nociceptive neurons.^10^ Likewise, the TRPV4 antagonist HC067047 has shown to effectively reduce hypersensitivity in CP in mice.^90^

The TRP family of channels also includes another prominent “nocisensitive” molecule, TRPA1, whose role in CP has been highlighted by 2 recent findings.^9,68^ First, animal models that are either TRPA1 knock-outs or employ TRPV1 antagonists demonstrate reduced fibrosis, inflammatory infiltrates, and neural hypertrophy, the histological hall marks of CP.^68^ Second, diminished TRPA1 and/or TRPV1 activity is associated with a reduction in sensitization to pain.^9^

Given this convincing evidence of enhanced baseline and evoked activity in pancreatic nociceptors (peripheral sensitization), it is then also important to try and identify the biological factors that are driving this sensitization. Several plausible candidates have been identified of which perhaps the most prominent is nerve growth factor (NGF). This is normally only expressed in pancreatic islets but with the development of CP becomes much more widely seen in the parenchyma in both acini and ducts. In order to gain deeper insight into the pathomechanisms of NGF, the effect of anti-NGF treatment has been studied in various animal models of CP. Neutralizing antibodies to NGF can not only treat pancreatic hyperalgesia in rats with CP but also prevent the changes in I_*A*_ potassium currents and associated hyperexcitability of pancreatic nociceptors.^93^ Further, anti-NGF treatment in rats can also counteract the increase in TRPV1 currents and expression, as well as SP and CGRP in these neurons.^52,94^ Another cell type that produces NGF is the mast cell, whose numbers are increased in painful but not painless CP.^39^ Further, mast cells are also a source of tryptase, which along with trypsin can enhance TRPV1 currents via the PAR-2 receptor. Other neurogenic mediators such as SP can in turn degranulate mast cells, leading to perpetuation of the pro-nociceptive state. In accordance with an important role for these cell types, pain responses in mast cell deficient mice with CP are significantly reduced.^39^

Another major contender for a “master molecule” for peripheral sensitization is transforming growth factor beta (TGFβ), which is known to be upregulated in the pancreas with chronic inflammation in both patients and rodent models.^46,91^ Treatment of sensory neurons with TGFβ in vitro induces changes in excitability and suppressed a specific voltage-dependent potassium (IA) current, which is a hallmark of nociceptive excitability in CP as discussed above.^92^ Neutralization of TGFβ in rats with CP can reverse pancreatic hyperalgesia. Given its classical role in promoting fibrosis, TGFβ signaling may prove to be a very important unifying mechanism to explain both pain and fibrosis, the 2 hallmarks of CP.

Yet as intriguing as the process of peripheral sensitization might be, the full extent of pancreatic pain still seems insufficiently evidenced. It is therefore necessary to take a closer look at reciprocal immuno-neuronal interactions and the characteristic long-term neuroplastic alterations they entail.

### 2.2. Peripheral pancreatic neuropathy: a vicious circle?

The 2 most common exocrine disorders of the pancreas, CP and pancreatic ductal adenocarcinoma, create a very similar inflammatory microenvironment, which is considered to be a breeding ground for neuronal impairment. Pancreatic neuropathy comprises 4 key aspects: (1) *pancreatic neuroplasticity*, (2) *pancreatic neuritis*, (3) *neural remodeling*, and (4) *neural invasion*.^14,17^ We previously demonstrated that all 4 characteristics strongly correlate with the intensity of neuropathic pain.^11^

In the course of disease, nerves stretching through the pancreatic tissue typically create a dense network of hypertrophic nerves. Corresponding histomorphological changes, ie, the increase in nerve diameter and neural density, are commonly called as “*pancreatic neuroplasticity*.”^17^ In this context, growth associated protein 43 (GAP 43) and Neurturin, 2 molecules supporting neuronal development and growth, are suspected to be substantially involved. In the past, the role of GAP 43, a neuronal plasticity marker normally expressed during the differentiation and regeneration of axons,^3^ has been extensively scrutinized. Various studies have associated GAP 43 overexpression in CP tissues with an increased degree of abdominal pain.^69^ Some recent findings shed light on the Neurturin/GFRa2 axis and its influence on pancreatic neuroplasticity. Neurturin belongs to the glial cell line–derived neurotrophic factor family of ligands and is known to orchestrate the normal development of parasympathetic innervation.^65^ To investigate the expression of neurturin and its receptor GFRa2 in CP tissue compared to normal pancreas, we applied a variety of in vitro methods and subsequently correlated our results to clinical pain reports. Neurturin and GFRa2 were upregulated in intrapancreatic nerves, particularly those surrounded by clusters of inflammatory cells, with a further upregulation in CP tissues. Interestingly, Neurturin presented as a dimer at 55 kDa in CP compared to its monomeric pro-form near 25 kDa in controls. To reach their biologically active form, inactive Neurturin monomers have to congregate in a multi-step process, eventually metamorphosing into tetramers or higher forms of multimers.^77^ This supports the idea that multimeric active forms of Neurturin are highly upregulated in CP tissue. With a novel neuroplasticity assay, we quantified the extent of neuroplastic alterations initiated by the Neurturin/GFRa2 axis. Rat dorsal root ganglia were cultured under different conditions and neurotrophic effects measured by the extent of neuronal density and phenotypic alterations. Although Neurturin expression was not correlated to neuropathic pain behavior, it strongly stimulated branching and neurite density in dorsal root ganglia neurons, leading to the conclusion that the neurturin/GFRa2 axis is a key player in pancreatic neuroplasticity.^17,20^

Apart from changing their phenotype, nerves often exhibit extended damage to the perineurium as their natural barrier, allowing the free entrance of lytic enzymes and inflammatory cells.^69^ Based on the close immuno-neuronal communication, the extent of “*perineural invasion and neuritis*” positively correlates with the degree of neuropathic alterations and pancreatic pain.^17^ Infiltrated fibers start to produce increased amounts of SP, a signaling molecule that not only paves the way for peripheral sensitization but also attracts an armada of leucocytes to the injured nerve. In turn, arriving immune cells release the cytokine IL8, which amplifies chemoattractive effects and has shown to be overexpressed in CP.^70^ Another hallmark of immuno-neuronal interaction is the chemokine Fractalkine and its receptor CX3CR1. Upregulated in chronic inflammatory tissues, Fractalkine is involved in glial activation, tissue fibrosis, and inflammatory response.^13^ Blockage of the receptor CX3CR1 could thus be a promising therapeutic approach for the treatment of pancreatic pain.^15^ If one takes a closer look at the tightly packed foci of immune cells clustering intrapancreatic nerves, they exhibit a strikingly characteristic distribution pattern: the majority is represented by CD8^+^ lymphocytes, followed by CD68^+^ macrophages, and mast cells and in particular the latter show a strong association with the degree of neuropathic pain.^18^

Another main feature of pancreatic neuropathy is the compositional change of nerve fibers, also known as “*neural remodeling*.” In CP, patients with extensive abdominal pain often exhibit a decreased innervation to the pancreas.^19^ This profound alteration of the nervous system not only involves neurons and their projections but also glia cells as their “cellular protectors.” Once stimulated, glia cells increasingly express the neuroepithelial stem cell marker Nestin, which can be regarded as a sign of activation after nerve injury. However, SOX10, the transcription factor usually presented by mature glia cells, is downregulated.^12^ Although CP and human pancreatic adenocarcinoma both create similar inflammatory microenvironments, characteristic changes in glia cell activity are even more pronounced in cancerous tissues. In human PDAC, glia cells are thought to have an almost “magnetic” affinity to their malignant counterparts. The myelin-associated glycoprotein/MAG serves as a glial receptor for the cancer cell surface antigen Mucin1, allowing the adhesion and mutual binding of both cells.^71^ Glia cells are even believed to pave the way towards malignancy, as they are often encountered in a neatly arranged fashion around early precursor lesions.^16^ This “*Schwann cell carcinotropism*” is mainly mediated by NGF, produced by cancer cells, which allows them to attach to the p75NTR receptor on Schwann cells.^17^ As neurons and PCa cells subsequently lie near each other, malignant cells can easily penetrate the perineurium and infiltrate the nerve. In the context of “*perineural invasion*,” cancer cells express increasing amounts of NGF, Artemin, and Neurturin.^17^ Those neurotrophic factors not only fuel the process of perineural invasion but also contribute to the sensitization of peripheral nerve endings.^17^ At this point, the neuropathic aspect of pancreatic pain is probably most vividly displayed. Chronically impaired nerves are not only damaged by the disease itself but also by the complex interplay of neurons, glia, inflammatory and cancer cells, locked in a vicious circle of mutual reinforcement.

According to the IASP, neuropathic pain is defined as a “pain that arises as a direct consequence of a lesion or diseases affecting the somatosensory system” (https://www.iasp-pain.org/GlobalYear/NeuropathicPain). This novel characterization of neuropathic pain encompasses histopathologic changes (“lesions”) and autoimmune and inflammatory processes (“disease”).^73^ As outlined in the sections above, the microenvironment of CP meets both conditions by inducing secondary neuroplastic alterations in pancreatic neuropathy, as well as an upregulation of peripheral nociception following chronic inflammation. Therefore, CP pain is neither solely nociceptive, nor neuropathic, but can be understood as a “mixed-type” sensation.^19^

### 2.3. Central neuroplasticity: cause or consequence of pancreatic pain?

An increased afferent neuronal barrage to the spinal cord may result in an increased responsiveness of central pain transmitting neurons. This phenomenon is known as *central sensitization* and refers to an increased synaptic efficacy established in sensory neurons in the dorsal horn of the spinal cord following intense peripheral noxious stimuli, tissue injury, or nerve damage.^49^ Ultimately, ongoing peripheral stimulation may result in a long-lasting increase in the excitability of spinal cord neurons, profoundly changing the gain of the sensory system, where the pain processing is no longer coupled to the presence, intensity, or duration of noxious peripheral stimuli. Various mechanisms have been associated with central sensitization that typically comprise 2 temporal phases: (1) an early phosphorylation-dependent and transcription-independent phase, which results mainly from rapid changes in glutamate receptor and ion channel properties and (2) a later, longer lasting, transcription-dependent phase, which drives synthesis of new proteins responsible for the longer-lasting form of central sensitization observed in different pain conditions.^83^ One of the best characterized mechanisms in the early phase of central sensitization is activation of the N-methyl-d-aspartic acid (NMDA) receptor, revealing a key involvement of glutamate in this process.^80^ Blocking of the NMDA receptor by ketamine was shown to reverse hyperalgesia associated with CP in an experimental study.^6^

Central sensitization manifests as a reduction in pain thresholds with pain in response to a non-noxious stimulus (allodynia), an increase in responsiveness and prolonged aftereffects to noxious stimuli (hyperalgesia), and a receptive field expansion that enabled input from non-injured tissue to produce pain (secondary hyperalgesia) (Table 1).^82^ Several studies have reported findings compatible with central sensitization in CP. In one study, increased areas of referred pain to electrical stimulation of the esophagus, stomach, and duodenum was reported in patients with CP compared to control subjects.^21^ Other studies reported decreased pain thresholds to visceral stimulation of the rectosigmoid as well as somatic stimulation of muscle and bone,^8,56^ and hyperalgesia seemed to be linked to disease severity in patients with CP.^7^ In addition, the classical post-prandial worsening of pain seen in many patients with CP may represent allodynia triggered by the passage of food through the upper gut and stimulation of the pancreas.^24^ Taken together, these findings characterize a generalized hyperalgesic state of the pain system and likely mirrors widespread sensitization of the central sensory pathways as seen in many other chronic pain disorders.^82^

**Table 1:**
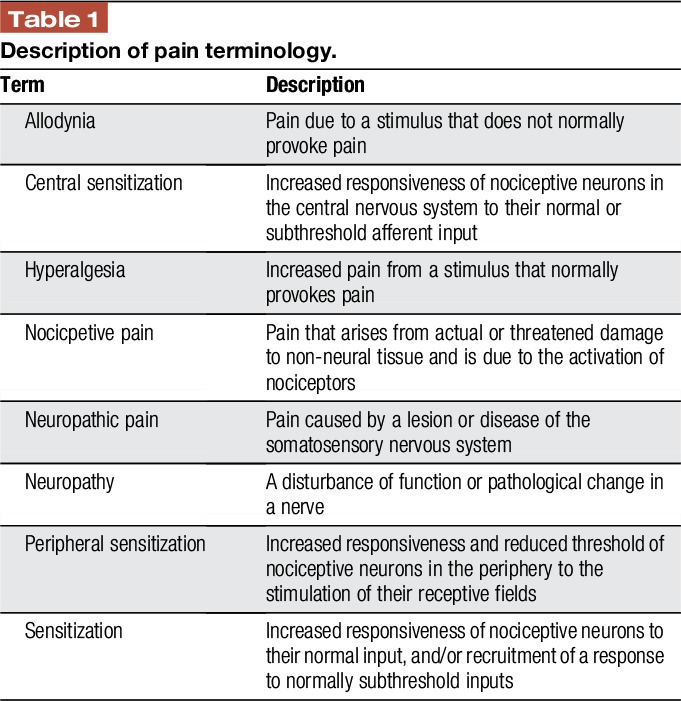
Description of pain terminology.

Several experimental and clinical studies have indicated that peripheral neuropathy and neural deafferentation is associated with a functional reorganization of the cerebral cortex.^28^ For example, people with arm or hand amputations show a shift of the mouth into the hand representation in the primary somatosensory cortex, with the quantity of cortical reorganization being correlated with subjective pain ratings.^27^ In patients with CP, the pancreatic neuropathy discussed above may to some degree mimic the peripheral nerve pathology seen in patients following amputations. Along this line, experimental pain studies, based on somatic stimulation of the skin area in the Th10 dermatome (sharing spinal segmental innervation with the pancreatic gland) as well as visceral stimulation of the upper and lower gut with concomitant recording of evoked brain potentials and brain source localization, have indicated that chronic pain and hyperalgesia are associated with functional reorganization of the cortical areas involved in visceral pain processing.^21,50,57^ Hence, compared to healthy controls, patients with CP demonstrated reorganization in the insula, secondary somatosensory cortex, and cingulate cortex. In addition, the excitability of these neural networks was abnormal with evidence of impaired habituation to noxious stimuli, possibly reflecting a cortical neuronal hyperexcitability (ie, cortical sensitization).^60^ Finally, the thalamus, as a critical relay site in the sensory system, has been implicated in chronic pain; a disturbance of the thalamocortical interplay seen as global changes in the rhythmicity of the cerebral cortex was observed in patients with neuropathic pain of mixed origin.^66^ Parallel findings were observed in patients with CP in studies based on spectral analysis of visceral evoked brain potentials and resting state electroencephalography.^22,59^

The structural correlate of functional cortical reorganization and hyperexcitability is found in studies based on structural magnetic resonance imaging (MRI). In a CP study, using diffusion weighted MRI, microstructural changes in the insular and frontal brain areas were associated with clinical pain intensity and functional scores.^32^ Patients with a constant pain pattern demonstrated the most severe microstructural abnormalities compared to patients with an attack-wise pain pattern, which translates well to the clinical situation where patients with constant pain are reported to have the most reduced quality of life.^54^ In another MRI study based on cortical volumetric assessment, brain areas involved in visceral pain processing was shown to have a reduced thickness.^30^ These findings attest to the neuroplastic changes observed in painful CP.

The sensory system has several inherent mechanisms whereby inflowing neuronal signals are modulated. Descending modulatory pathways from the brain stem and higher cortical structures play a key role in such endogenous sensory modulation and control the afferent input of neuronal signals at the spinal level. The process can lead to either an increase in the spinal transmission (facilitation) or a decrease in transmission (inhibition) and is mediated through distinct dopaminergic, serotonergic, adrenergic, and opioidergic pathways. The balance between these pathways ultimately determines the quality and strength of the neuronal signals entering spinal transmission and cortical processing.^38^ Alterations in the state of descending modulation from inhibition towards facilitation have been implicated in the transition of acute into chronic and neuropathic pain. Thus, several studies, both animal and human, have documented the involvement of brainstem structures in the generation and maintenance of central sensitization and hyperalgesia.^34,89^ In the context of pain and CP, impaired descending inhibitory pain modulation have been reported in studies based on the conditioned pain modulation paradigm, where descending modulation was induced experimentally by applying a prolonged tonic pain stimulus (conditioning stimulus—cold-pressor test) and quantified by applying a test-pain stimulus (quadriceps pressure stimulation) before and after its induction.^7,56^ Also, in a rat model of CP, the persistence of pancreatitis pain was dependent on descending pain faciliatory mechanisms arising in the rostral ventromedial medulla with subsequent upregulation of spinal dynorphin.^76^

**Figure 3.**
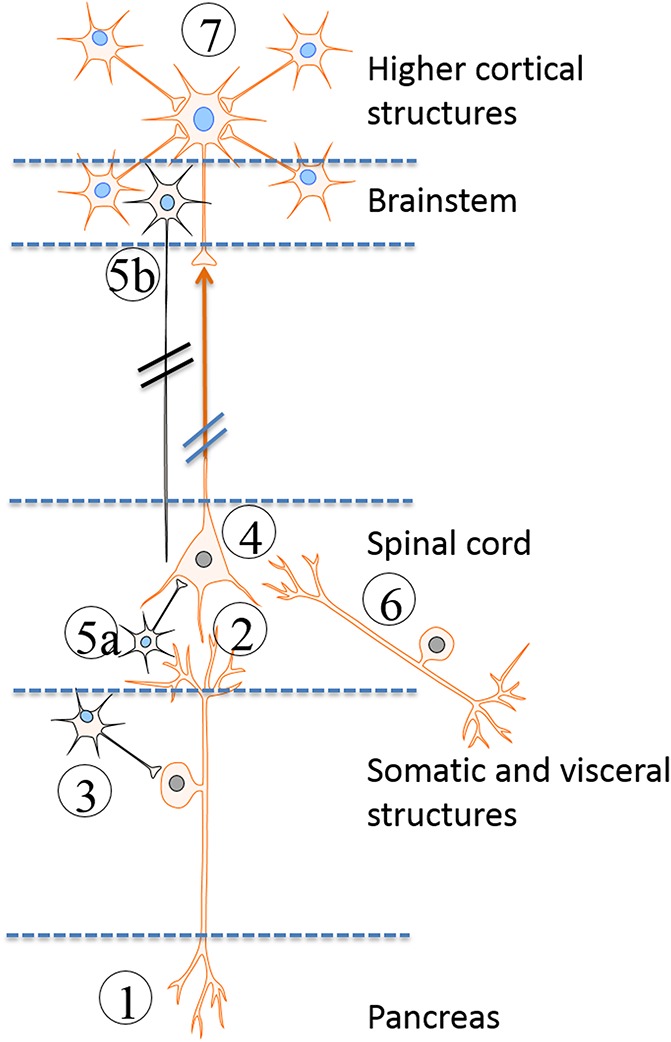
Schematic illustration of the different neurobiological mechanisms involved in pancreatic pain: (1) Peripheral nerve damage in the pancreas with ectopic activity resulting in stimulus-dependent and spontaneous pain. (2) Sprouting of non-nociceptive nerve afferents into areas of the spinal cord that normally transmit nociceptive information resulting in allodynia. (3) Sprouting of sympathetic neurons (black) into the dorsal horn neurons rendering the system sensitive to sympathetic activity. (4) Sensitisation and phenotypic changes of spinal neurons due to the increased afferent barrage. (5) Defects in the normal inhibition of the incoming nociceptive information from (a) interneurons and (b) descending tracts arising in the brainstem (black). (6) Abnormal coding of the afferent input from somatic areas and other viscera resulting in increased referred pain areas and viscero-visceral hyperalgesia. (7) Reorganisation and structural changes in the brain that encodes complex sensations such as affective, evaluative, and cognitive responses to pain.

Taken together and as outlined above, several lines of evidence indicate that central neuroplasticity are truly present in CP (Fig. 3). However, from the current evidence it is difficult to determine whether these central changes are maintained by a sustained nociceptive drive from the pancreatic gland (ie, a *consequence* of pancreatic pain) or whether they have become independent of peripheral stimuli and thus comprise an autonomous and self-perpetuating state of the sensory system that may generate “pain on its own” (ie, a *cause* of pancreatic pain).^35^ In favor of the latter, a small cross-sectional study found that generalized hyperalgesia was associated with failure of thoracoscopic splanchnic denervation.^5^ The authors proposed that in hyperalgesic patients the generation of pain had become independent of the initial peripheral nociceptive drive and consequently denervation of peripheral nerves was ineffective. However, in somatosensory disorders, such as peripheral nerve injury and painful polyneuropathy, there is support that regardless of signs of central sensitization, a primary afferent input is critical for maintaining ongoing and evoked neuropathic pain.^37,75^ The efficacy of topically applied drugs in these conditions also supports that peripheral pain-generating mechanisms are key to maintain central sensitization and hyperalgesia.^5^

## 3. Implications for treatment

A thorough discussion of endoscopic, surgical, and medical pain treatment in CP is beyond the scope of this article and the reader is referred to recent published guidelines.^23,53^ The following treatment recommendations are based on the neurobiological understanding of pain in CP as outlined in the previous sections.

Many patients with chronic pain develop depression and if present antidepressive treatment may also improve pain.^33^ Patients with CP also report high rates of depression,^2^ and these cases can be identified through screening with questionnaires such as the Hospital Anxiety and Depression Scale.^4^ Although not documented in CP, patients with chronic pain and depression particularly benefit from antidepressant drugs from the SNRI class, such as venlafaxine, that has less adverse effects than tricyclic antidepressives and lowers pain in clinical studies of functional visceral pain.^74^ From a mechanistic point of view, these drugs act in the serotonergic and noradrenergic systems and facilitate descending inhibition and, as such, may theoretically benefit patients with CP.^56,88^

Adjuvant therapy with gabapentinoids, such as pregabalin, has been shown effective in patients with pain due to CP in a randomized placebo-controlled trial.^55^ Unfortunately, many patients suffer from adverse effects, including dizziness and lightheadedness, which may limit its use in the clinical setting. A mechanism-based guidance can select responders to pregabalin treatment, as those with evidence of central sensitization, evident as relative hyperalgesia in the Th10 dermatome (where neuronal afferents from the pancreas and somatic tissue converge), have a higher likelihood for effective treatment outcome.^58^

In addition to conventional treatment strategies, neuromodulation and other complementary treatment modalities may be useful in CP. Hence, spinal cord stimulation, transcranial magnetic stimulation, and acupuncture have all been proven effective in proof-of-concept studies, but rigorous evaluation in properly designed clinical trials have not yet been conducted.^29,43,44^ Neurolytic procedure such as endoscopic guided plexus blocks was previously widely used for pain in CP but is now considered obsolete due to poor long-term outcomes and risk of side effects such as postural hypotension and diarrhea.^78^ Thoracoscopic splanchnicectomy has been described as an alternative and minimally invasive therapy for pancreatic pain. However, pain relieving effects are short lasting and sham controlled studies have not been done.^42^ Taken together, the lack of effectiveness of these neurolytic procedures are likely explained by the associated nerve damages that may further worsen the pathological pain processing in peripheral and central nerves.

Many other treatments have been used to treat pain in patients with CP. Among them, psychological interventions such as cognitive behavioral therapy and hypnotherapy have been shown effective in various chronic pain conditions.^45^ Along this line, in a recent case series we showed that hypnotherapy was effective for pain in CP (Juel J et al. in preparation 2017). Although large-scale clinical studies are needed to further document the efficacy of psychological intervention in CP, it is generally believed that the psychosocial dimension of pain are comparable between pain conditions, which rationalizes that psychological intervention should also be offered to patients with CP.

## 4. Conclusion

Research over the last decade has improved our understanding of pain mechanism in CP substantially and several lines of evidence support a neurobiological origin of pain that in many cases resembles that observed in neuropathic pain conditions. This improved understanding of pain has important clinical implications for treatment; adjuvant analgesics, as well as neuromodulation and psychological interventions, may prove useful in the future but need further validation.

## Disclosures

The authors have no conflicts of interest to declare.
